# Predicting progression from mild cognitive impairment to Alzheimer’s disease on an individual subject basis by applying the CARE index across different independent cohorts

**DOI:** 10.18632/aging.101883

**Published:** 2019-04-30

**Authors:** Jiu Chen, Gang Chen, Hao Shu, Guangyu Chen, B. Douglas Ward, Zan Wang, Duan Liu, Piero G. Antuono, Shi-Jiang Li, Zhijun Zhang, Alzheimer’s Disease Neuroimaging Initiative

**Affiliations:** 1 Department of Neurology, Affiliated ZhongDa Hospital, School of Medicine, Southeast University, Nanjing 210009, Jiangsu China; 2 Institute of Neuropsychiatry, the Affiliated Brain Hospital of Nanjing Medical University, Fourth Clinical College of Nanjing Medical University, Nanjing 210029, Jiangsu China; 3 Department of Biophysics, Medical College of Wisconsin, Milwaukee, WI 53226, USA; 4 Department of Neurology, Medical College of Wisconsin, Milwaukee, WI 53226, USA; 5 Department of Psychology, Xinxiang Medical University, Xinxiang 453003, Henan China; 6 A complete listing of ADNI investigators can be found at http://adni.loni.usc.edu/wp-content/uploads/how_to_apply/ADNI_Acknowledgement_List.pdf

**Keywords:** Alzheimer’s disease, mild cognitive impairment, Characterizing Alzheimer’s disease Risk Events (CARE) index, Alzheimer’s Disease Neuroimaging Initiative (ADNI)

## Abstract

The purposes of this study are to investigate whether the Characterizing Alzheimer’s disease Risk Events (CARE) index can accurately predict progression from mild cognitive impairment (MCI) to Alzheimer’s disease (AD) on an individual subject basis, and to investigate whether this model can be generalized to an independent cohort. Using an event-based probabilistic model approach to integrate widely available biomarkers from behavioral data and brain structural and functional imaging, we calculated the CARE index. We then applied the CARE index to identify which MCI individuals from the ADNI dataset progressed to AD during a three-year follow-up period. Subsequently, the CARE index was generalized to the prediction of MCI individuals from an independent Nanjing Aging and Dementia Study (NADS) dataset during the same time period. The CARE index achieved high prediction performance with 80.4% accuracy, 75% sensitivity, 82% specificity, and 0.809 area under the receiver operating characteristic (ROC) curve (AUC) on MCI subjects from the ADNI dataset over three years, and a highly validated prediction performance with 87.5% accuracy, 81% sensitivity, 90% specificity, and 0.861 AUC on MCI subjects from the NADS dataset. In conclusion, the CARE index is highly accurate, sufficiently robust, and generalized for predicting which MCI individuals will develop AD over a three-year period. This suggests that the CARE index can be usefully applied to select individuals with MCI for clinical trials and to identify which individuals will convert from MCI to AD for administration of early disease-modifying treatment.

## INTRODUCTION

Mild cognitive impairment (MCI) has been conceptualized as a transitional clinical state between normal and Alzheimer’s disease (AD)-type dementia [1]. MCI has been considered as a key prognostic and therapeutic target in the management of AD. However, not all MCI subjects convert to AD, and many individuals remain cognitively stable or revert to normal status [2]. Early detection of those progressive MCI individuals is of increasing clinical importance in the enrichment of clinical trials of disease-modifying therapies [3].

Building an effective and accurate prognostic model that predicts the progression from MCI to AD is clinically important. To this end, a large number of studies have combined magnetic resonance imaging (MRI)-based features with positron emission tomography (PET) [4]; cerebrospinal fluid (CSF) [5]; PET and CSF [4, 6]; assessment of cognitive function (CF) and CSF [7]; and CF, PET, and CSF measures [8, 9].

Significant progress has been made using machine learning tools. Most of the above-mentioned studies have integrated CSF and PET measures. This approach using multiple technologies (CF, CSF, PET, and MRI) is often not available in clinical practice. In particular, while the prediction performance of these models has been validated extensively in a nested cross-validation loop [10] and the traditional leave-one-out approach [11], they have not been validated in an independent population. This will most likely lead to biased results and over-optimistic accuracies [12]. Although there exist some methods using the outer cross-validation loop to evaluate generalizability [13], these have limited predictability in differentiating individuals with MCI who convert to AD from non-converters. However, an effective, accurate, and clinically useful prognostic model should be robust and generalized for both the routine clinical setting and drug trials.

Recent work has begun to address this limitation. Using cortical thickness regions and subcortical volumes, Westman and colleagues combine Alzheimer’s Disease Neuroimaging Initiative (ADNI) data with the AddNeuroMed dataset [14] to predict progression of MCI to AD [15]. However, they report a low robustness with accuracies of 58% in ADNI data and 70% in AddNeuroMed data. Using MRI, PET, and CSF biomarkers, Prestia and colleagues combined ADNI data with the Translational Outpatient Memory Clinic (TOMC) dataset to predict MCI progression to AD [6]. They also obtained low accuracies with ranges of 49–63% and 51–75% in ADNI and TOMC, respectively. Thus, for the early detection of AD progression, the accuracy of predicting progression from MCI to AD needs to be improved using sensitive, generalizable, widely available, cost-effective, and minimally invasive tools.

**Figure 1:**
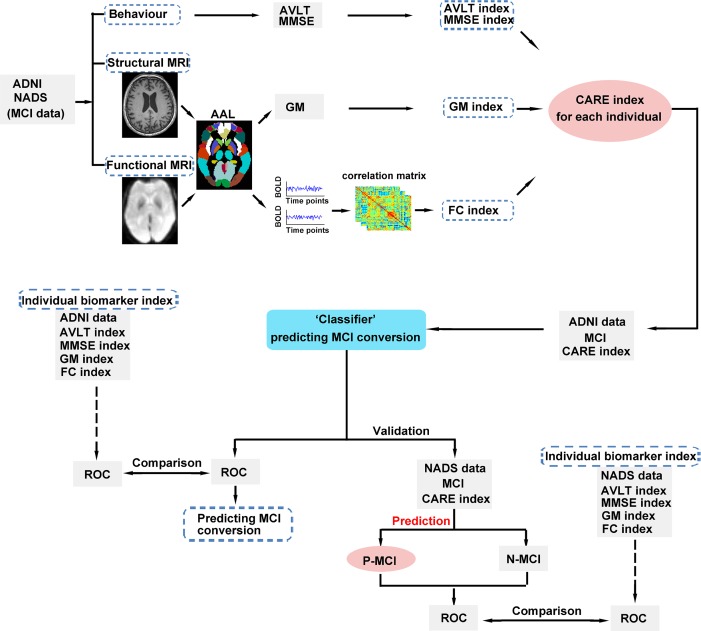
**Schematic diagram of the proposed classification, independent validation, and prediction framework.** First, we calculated each individual CARE index score for MCI subjects in the ADNI and NADS datasets. Second, we utilized a CARE index score (A score on the CARE index is equivalent to the subject’s disease stage.) to classify N-MCI and P-MCI subjects in the ADNI dataset. Third, we applied the ‘CARE index stage “classifier” determined from the ADNI dataset to predict the conversion of MCI subjects in the NADS dataset. The ROC curve was used to assess the performance of the CARE index stage classifier and the CARE index stage prediction classifier, respectively. In addition, we assessed the performance differences of CARE index stage classification and prediction and original indices (AVLT, MMSE, GM, and FC indices) by comparing these ROCs across datasets. Abbreviations: MCI, mild cognitive impairment; ADNI, Alzheimer’s Disease Neuroimaging Initiative; NADS, Nanjing Aging and Dementia Study; AD, Alzheimer’s disease; MMSE, Mini-Mental State Examination; AVLT, Rey Auditory Verbal Learning Test; MRI, magnetic resonance imaging; AAL, automated anatomical labeling; GM, grey matter; BOLD, blood oxygenation level dependent; FC, functional connectivity; CARE, characterizing AD risk event; ROC, receiver operating characteristic; P-MCI, progressive MCI, including MCI subjects who progressed to AD-type dementia at the three-year follow-up; N-MCI, no-progressive MCI, including MCI subjects who had not progressed to dementia at the three-year follow-up.

Here, we will address this challenge with our newly developed framework, termed the characterizing AD risk events (CARE) index [16]. The CARE index estimates the probabilities of occurrence and nonoccurrence of a series of biomarkers by using an event-based probabilistic model [16]. In our previously published study, the CARE index was proven to accurately stage each individual across the whole AD spectrum [16]. It is of crucial importance to note that predicting the conversion over a short future time is an easier problem than over a longer one and less clinically useful (Eskildsen et al., 2013; Young et al., 2013), and means a smaller proportion of MCI subjects will likely convert to AD at a later time, which reduces the positive predictive value of the classification result (Young et al., 2013). Therefore, we have chosen a three-year follow-up period. In this study, we applied the CARE index to the ADNI dataset to distinguish those individuals with MCI who progressed to AD from those who did not, during a three-year follow-up period. Subsequently, we generalized the CARE index to predict which MCI from the independent Nanjing Aging and Dementia Study (NADS) dataset would convert to AD during the same time period (Figure 1). Based on the previous literature [16], We hypothesized that the CARE index score accurately predicted MCI-to-AD progression with high sensitivity and specificity at the individual patient level over 3 years in the ADNI dataset. We further hypothesized that the excellent prediction performance of the CARE index could be validated in the independent NADS dataset and achieve good between-cohort generalization during the same time-period.

## RESULTS

### Baseline demographic and neuropsychological characteristics

The demographic and neuropsychological characteristics of the included groups are listed in Table 1. As expected, in the ADNI dataset, N-MCI and P-MCI showed no significant differences in age, gender, education, and MMSE scores (p > 0.05). In the NADS dataset, P-MCI showed no significant differences in gender and education (p > 0.05), but higher age and lower MMSE scores than N-MCI (p < 0.05). Compared with N-MCI, P-MCI showed significant deficits in performance in multiple domains of cognitive functions, including episodic memory, information processing speed, and executive function (all p < 0.05). Furthermore, there was no difference in the conversion rates of different MCI state transitions between the ADNI and NADS datasets (*χ*2 = 0.078, *p* = 0.78, see Supplementary Table 1).

**Table 1:** Demographics and clinical measures of N-MCI and P-MCI subjects at baseline.

**Item**	**N-MCI**	**P-MCI**	***t* value (χ2)**	***p* value**
	**n=34**	**n=12**		
**ADNI data**				
Age (years)	70.24 (7.20)	73.90(5.41)	-1.605	0.116
Gender (male/female)	17/17	6/6	0.000	1.000
Education level (years)	15.76(2.73)	15.75(2.45)	0.016	0.987
MMSE	27.71(1.77)	27.42(1.38)	0.513	0.610
ADAS-Cog	8.62(3.23)	13.08(4.21)	-3.800	0.000*
AVLT	37.47(10.20)	29.25(5.85)	3.382	0.002*
**Item**	**N-MCI**	**P-MCI**	***t* value (χ2)**	***p* value**
	**n=40**	**n=16**		
**NADS data**				
Age (years)	66.73(7.05)	72.93(5.79)	-3.123	0.003*
Gender (male/female)	23/17	9/7	0.007	0.932
Education level (years)	11.93(3.48)	11.38(2.94)	0.557	0.580
MMSE	27.53(1.60)	24.56(2.63)	5.154	0.000*
**Composite Z scores of each cognitive domain**				
Episodic memory	0.33(0.57)	-0.82(0.73)	6.338	0.000*
Information processing speed	0.17(0.73)	-0.42(0.71)	2.780	0.007*
Executive function	0.22(0.52)	-0.56(0.49)	5.132	0.000*
Visuospatial function	0.05(0.52)	-0.13(0.52)	1.148	0.255

Notes: Values are expressed as the mean (standard deviation, SD). * Significant differences are found between P-MCI and N-MCI subjects. *P* values are obtained by *t* test except for gender (chi-square test). The performances of MMSE are presented as raw scores. The level of each cognitive domain is denoted by the composite Z scores.

Abbreviations: MCI, mild cognitive impairment; MMSE, Mini-Mental State Examination; ADNI, Alzheimer’s Disease Neuroimaging Initiative; NADS, Nanjing Aging and Dementia Study; P-MCI, progressive MCI, including MCI subjects who have progressed to AD-type dementia at the three-year follow-up; N-MCI, nonprogressive MCI, including MCI subjects who have not progressed to dementia at the three-year follow-up. ADAS-Cog, Alzheimer’s Disease Assessment Scale-Cognitive Subscale; AVLT, Rey Auditory Verbal Learning Test

### Discriminating N-MCI/P-MCI

**Figure 2:**
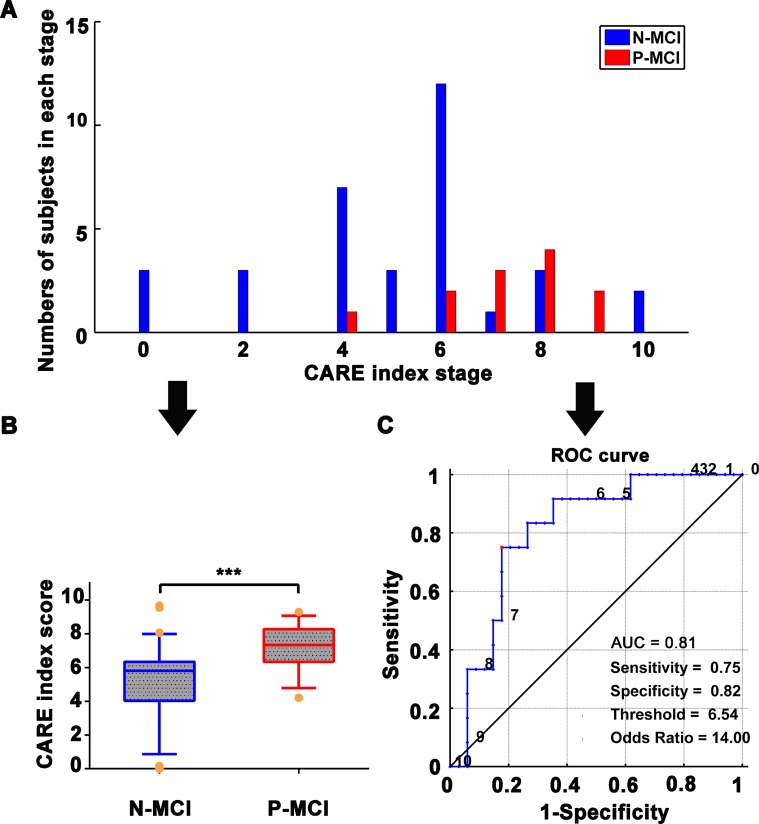
**Classification of N-MCI and P-MCI in the ADNI dataset by the CARE index.** (**A**) Number of patients in each diagnostic category at each CARE index stage at baseline; data from the ADNI dataset. N-MCI subjects are represented in blue and P-MCI subjects in red. Each CARE index stage on the *x*-axis corresponds to the occurrence of a new biomarker transition event. Stage 0 corresponds to no events having occurred and stage 10 to all events having occurred. The optimal temporal sequence, S^optimal^, of the 10 AD Biomarkers was used to calculate the CARE index. The S^optimal^ was estimated by the event-based probabilistic model. The S^optimal^ biomarker sequence is 1) increased HIP FCI, 2) decreased PCC FCI, 3) decreased Aβ concentration, 4) increased p-tau concentration, 5) decreased MMSE score, 6) increased ADAS score, 7) decreased HIP GMI, 8) decreased AVLT score, 9) decreased FG GMI, and 10) increased FG FCI. The details of the calculation of the S^optimal^ biomarker sequence and CARE index score can be found in our previously published studies [16] and are also provided in SI Methods. (**B**) Boxplot representing the distribution comparison of N-MCI and P-MCI subjects. For each boxplot, the band represents the median value, the box represents the interquartile range, and whiskers show the range of data without outliers (an outlier being defined as any value that lies more than one-and-a-half times the interquartile range from either end of the box). Differences were assessed between the two groups using Mann-Whitney tests; ***p < 0.001. (**C**) The power of receiver operating characteristic (ROC) curve of the CARE index “classifier” in classifying the diagnosis of P-MCI versus N-MCI at baseline in the ADNI dataset. Note: Numbers next to the ROC curve indicate the CARE index threshold. The values of sensitivity, specificity, and odds ratio in lower right of the figure present the optimum values under the optimum CARE index threshold (red piont). Abbreviations: ADNI, Alzheimer’s Disease Neuroimaging Initiative; AD, Alzheimer’s disease; P-MCI, progressive MCI, including MCI subjects who progressed to AD-type dementia at the three-year follow-up; N-MCI, nonprogressive MCI, including MCI subjects who had not progressed to dementia at the three-year follow-up; MCI, mild cognitive impairment; CARE, characterizing AD risk event; ROC, receiver operating characteristic; AUC, area under curve; Opt, optimum; HIP, hippocampus; PCC, posterior cingulate cortex; FG, fusiform gyrus; FCI, functional connectivity indices; GMI, gray matter indices; Aβ, β-amyloid; p-tau, phosphorylated tau; MMSE, Mini-Mental State Examination; ADAS-Cog, Alzheimer’s Disease Assessment Scale-Cognitive Subscale; AVLT, Rey Auditory Verbal Learning Test.

Both ADNI and NADS datasets showed that the CARE index differentiated P-MCI subjects from N-MCI subjects at baseline (see Figure 2). The comparative results on individual biomarker indices between N-MCI and P-MCI subjects are provided in Supplementary Figure 1.

As shown in Figure 2, the CARE index discriminated MCI progression to AD with an area under curve (AUC) of 0.81. The optimal CARE index threshold for discrimination between P-MCI and N-MCI subjects was found at a CARE index score of 6.54, with a sensitivity of 75.0%, specificity of 82.4%, odds ratio (OR) of 14.0, and relative risk (RR) of 6.20.

### Independent validation in different cohorts

**Figure 3:**
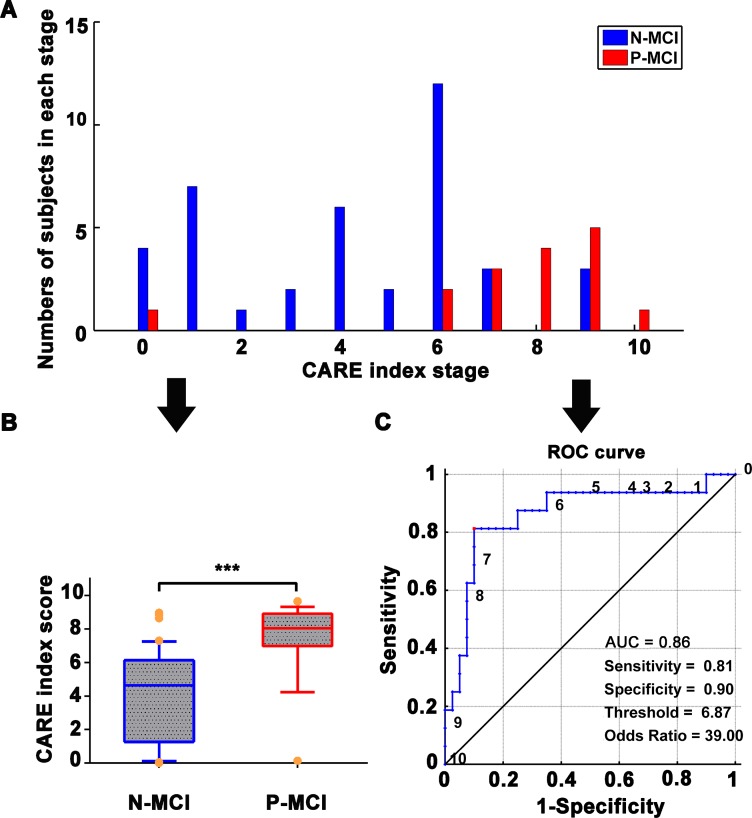
**Independent validation with NADS dataset for prediction of N-MCI and P-MCI subjects using the CARE index.** (**A**) Number of patients in each diagnostic category at each individual CARE index stage at baseline from the NADS dataset. N-MCI subjects are represented in blue and P-MCI subjects in red. (**B**) Boxplot representing the distribution comparison of N-MCI and P-MCI subjects. For each boxplot, the band represents the median value, the box represents the interquartile range, and whiskers show the range of data without outliers (an outlier being defined as any value that lies more than one and a half times the interquartile range from either end of the box). Differences were assessed between the two groups using Mann-Whitney tests; ***p < 0.001. (**C**) The power of the ROC curve of the CARE index “classifier” in predicting P-MCI versus N-MCI at baseline in the NADS dataset. Note: Numbers next to ROC curve indicate CARE index threshold. The values of sensitivity, specificity, and odds ratio in lower right of the figure present the optimum values under the optimum CARE index threshold (red piont). Abbreviations: NADS, Nanjing Aging and Dementia Study; AD, Alzheimer’s disease; P-MCI, progressive MCI, including MCI subjects who progressed to AD-type dementia at the three-year follow up; N-MCI, nonprogressive MCI, including MCI subjects who had not progressed to dementia at the three-year follow up; MCI, mild cognitive impairment; CARE, characterizing AD risk event; ROC, receiver operating characteristic; AUC, area under curve.

As shown in Figure 3, the CARE index had significant power to discriminate P-MCI subjects from N-MCI subjects, with an AUC of 0.86. At the CARE index of 6.87, the prediction has high sensitivity (81.3%), specificity (90.0%), and OR (39.0). When combining both the ADNI and NADS datasets on MCI subjects as a single cohort, supplemental analysis also showed that the CARE index discriminated P-MCI subjects from N-MCI subjects on an individual subject basis, with 83.3% accuracy, 82.0% balanced accuracy, 79% sensitivity, 85% specificity, AUC of 0.84 in receiver operating characteristic (ROC) curves on MCI subjects. (See Supplementary Figure 2, Supplementary Table 2.)

### Generalization in different cohorts

To validate the generalizability of the CARE index to discriminate P-MCI subjects from N-MCI subjects, we applied the optimal the CARE index threshold (6.54) to discriminate between the P-MCI and N-MCI subjects in the ADNI dataset and the MCI subjects in the NADS dataset. We found that, of the 56 MCI subjects, 48 (85.7%) were predicted correctly. MCI subjects with a CARE index score above the threshold have a high diagnostic odds ratio (OR = 33.33, 95% CI = 6.33–145.30) and relative risk (RR = 9.15, 95% CI = 2.98–28.13) in MCI progression relative to non-progression to AD. (See Table 2.)

**Table 2:** Estimated risk of the 3-year conversion to probable AD in MCI patients in NADS dataset using the optimal cutoff value of CARE index from ADNI dataset.

**Dataset**	**CARE index cutoff**	**Total**		**MCI**							
			**P-MCI**	**N-MCI**	**OR**	**95% CI for OR**	**RR**	**95% CI for RR**	**ACC**	**Sensitivity**	**Specificity**
ADNI											
	Converters (> = 6.54)	15	9	6							
	Nonconverters (< 6.54)	31	3	28	14.00	2.89–67.72	6.20	1.96–19.62	80.43%	75.0%	82.4%
NADS											
	Converters (> = 6.54)	18	13	5							
	Nonconverters (< 6.54)	38	3	35	30.33	6.33–145.30	9.15	2.98–28.13	85.71%	81.3%	87.5%

Note: 6.54 is the optimal cutoff value found using the ROC analysis to predict with optimal sensitivity and specificity those MCI patients from the ADNI dataset who did or do not convert to AD within three years.

Abbreviations: ADNI, Alzheimer’s Disease Neuroimaging Initiative; NADS, Nanjing Aging and Dementia Study; CARE, characterizing AD risk event; P-MCI, progressive MCI, including MCI subjects who progressed to AD-type dementia at the three-year follow-up; N-MCI, nonprogressive MCI, including MCI subjects who had not progressed to dementia at the three-year follow-up; MCI, mild cognitive impairment; AD, Alzheimer’s Disease; OR, odds ratio; RR, relative risk; CI, confidence interval; ACC, accuracy.

### Robustness and powerfulness of the CARE index compared to a single biomarker

**Figure 4:**
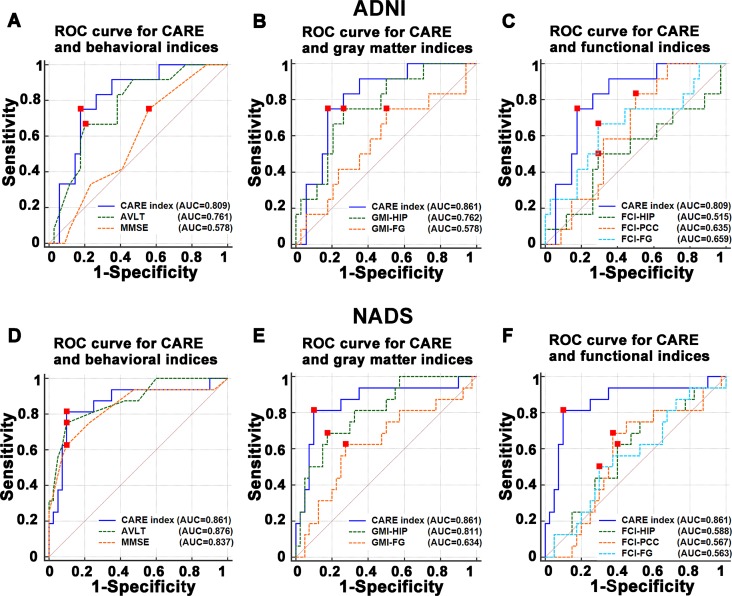
**Comparisons of the power of ROC curve of the CARE index with behavioral, gray matter, and functional indices in predicting the P-MCI versus N-MCI subjects in the ADNI and NADS datasets.** (**A**–**C**) represent comparisons of the power of ROC curve of the CARE index and individual behavioral, gray matter, and functional indices in the ADNI dataset, respectively. (**D**–**F**) represent comparisons of the power of ROC curve of the CARE index and individual behavioral, gray matter, and functional indices in the NADS dataset, respectively. Abbreviations: ADNI, Alzheimer’s Disease Neuroimaging Initiative; AD, Alzheimer’s disease; P-MCI, progressive MCI, including MCI subjects who progressed to AD-type dementia at the three-year follow up; N-MCI, nonprogressive MCI, including MCI subjects who had not progressed to dementia at the three-year follow up; MCI, mild cognitive impairment; CARE, characterizing AD risk event; ROC, receiver operating characteristic; AUC, area under curve; HIP, hippocampus; PCC, posterior cingulate cortex; FG, fusiform gyrus; FCI, functional connectivity indices; GMI, gray matter indices; Aβ, β-amyloid; p-tau, phosphorylated tau; MMSE, Mini-Mental State Examination; ADAS-Cog, Alzheimer’s Disease Assessment Scale-Cognitive Subscale; AVLT, Rey Auditory Verbal Learning Test.

As shown in Figure 4 and Table 3, in the ADNI dataset, the CARE index performed better than each of the seven selected biomarker indices in discriminating P-MCI subjects from N-MCI subjects. (See Figure 4 and Table 3). In the NADS dataset, although the best single biomarker index was an AVLT score with an AUC of 0.876, the CARE index power in discriminating P-MCI subjects from N-MCI subjects showed high generalization and stability across datasets, whereas other individual biomarkers did not. (See Table 3.)

**Table 3:** Sensitivity for P-MCI, specificity for N-MCI, AUC, and accuracy of CARE index and each of seven selected biomarker indices when applying the optimal threshold from the ADNI dataset to the NADS dataset.

**ADNI**	**Predictors**	**AUC**			**p**	**Sensitivity**		**Specificity**		**Accuracy**		**Balanced Accuracy**		**Opt. Threshold**
		**Rank**	**P-MCI + N-MCI**	**95% CI**		**Rank**	**P-MCI**	**Rank**	**N-MCI**	**Rank**	**%**	**Rank**	**%**	
	CARE index	**1**	**0.809**	**0.68-0.94**	**0.02**	**2**	0.75	**1**	0.82	**1**	80.4	1	78.7	6.54
	MMSE	**6**	**0.578**	**0.42-072**	**0.42**	**2**	0.75	**8**	0.44	**2**	76.1	8	59.6	28.5
	AVLT	**3**	**0.761**	**0.61-0.87**	**0.08**	**6**	0.69	**2**	0.79	**8**	52.2	3	73.0	28.5
	HIP^FCI^	**8**	**0.515**	**0.36-0.67**	**0.88**	**8**	0.50	**4**	0.71	**5**	65.2	7	60.3	0.66
	PCC^FCI^	**5**	**0.635**	**0.48-0.77**	**0.17**	**1**	0.83	**6**	0.50	**6**	58.7	5	66.7	2.28
	FG^FCI^	**4**	**0.659**	**0.51-0.79**	**0.10**	**7**	0.67	**4**	0.71	**4**	69.6	4	68.6	10.57
	HIP^GMI^	**2**	**0.762**	**0.61-0.88**	**0.01**	**2**	0.75	**3**	0.74	**3**	73.9	2	74.3	0.40
	FG^GMI^	**6**	**0.578**	**0.42-0.72**	**0.42**	**2**	0.75	**6**	0.50	**7**	56.5	6	62.5	0.55
**NADS**		**AUC**			**p**	**Sensitivity**		**Specificity**		**Accuracy**		**Balanced Accuracy**		**Opt. Threshold**
		**Rank**	**P-MCI + N-MCI**	**95% CI**		**Rank**	**P-MCI**	**Rank**	**N-MCI**	**Rank**	**%**	**Rank**	**%**	
	CARE index	**2**	**0.861**	**0.74-0.94**	**0.00**	**2**	0.81	**3**	0.88	**1**	85.7	1	**84.4**	6.54
	MMSE	**3**	**0.837**	**0.71-0.92**	**0.00**	**1**	0.94	**8**	0.32	**8**	50.0	4	63.1	28.5
	AVLT	**1**	**0.876**	**0.74-0.95**	**0.00**	**3**	0.75	**2**	0.90	**1**	85.7	2	82.5	28.5
	HIP^FCI^	**6**	**0.588**	**0.45-0.72**	**0.31**	**4**	0.69	**7**	0.53	**7**	57.1	5	60.6	0.66
	PCC^FCI^	**7**	**0.567**	**0.43-0.70**	**0.44**	**8**	0.31	**5**	0.70	**6**	58.9	8	50.6	2.28
	FG^FCI^	**8**	**0.563**	**0.42-0.70**	**0.47**	**6**	0.38	**5**	0.70	**5**	60.7	7	53.8	10.57
	HIP^GMI^	**4**	**0.811**	**0.68-0.90**	**0.00**	**6**	0.38	**1**	0.95	**3**	78.6	3	66.3	0.40
	FG^GMI^	**5**	**0.634**	**0.50-0.76**	**0.12**	**5**	0.44	**4**	0.78	**4**	67.9	5	60.6	0.55

Abbreviations: ADNI, Alzheimer’s Disease Neuroimaging Initiative; NADS, Nanjing Aging and Dementia Study; CARE, characterizing AD risk event; P-MCI, progressive MCI, including MCI subjects who progressed to AD-type dementia at the three-year follow-up; N-MCI, nonprogressive MCI, including MCI subjects who had not progressed to dementia at the three-year follow-up; MCI, mild cognitive impairment; AD, Alzheimer’s Disease; AVLT, Rey Auditory Verbal Learning Test; MMSE, Mini-Mental State Examination; HIP, hippocampus; PCC, posterior cingulate cortex; FUS, fusiform gyrus; GM, gray matter; GMI, gray matter indices; FCI, functional connectivity indices; Opt, optima.

### Behavioral significance of the changes in CARE index measured at baseline and the three-year follow-up

**Figure 5:**
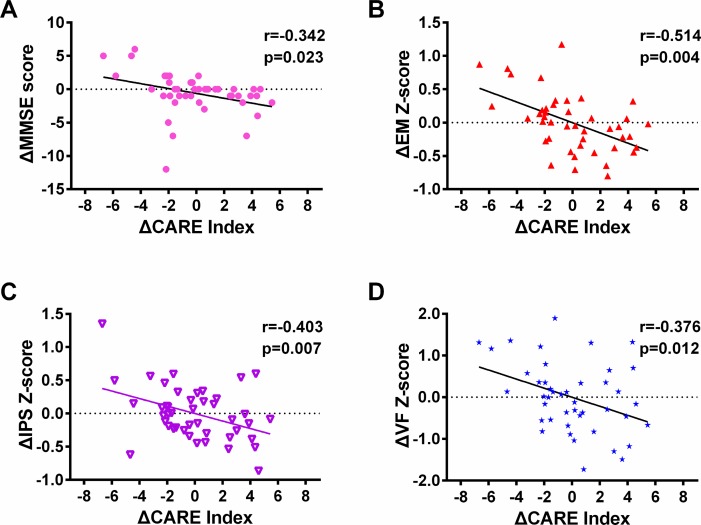
**The changes in the CARE index correlated with the changes in cognitive performance and other clinical variables measured between baseline and the three-year follow up in MCI subjects from the NADS dataset**. Notes: 1) In each of these scatter diagrams, there is a significant correlation between changes in the CARE index and changes in the cognitive performance or clinical measures (*p* < 0.05, FDR corrected). 2) In Figure 4, the total number of subjects selected at baseline is equivalent of those at the three-year follow-up from the NADS dataset (MCI, 44 subjects). All subjects at the three-year follow-up had at least one resting-state functional connectivity MRI scan with a corresponding anatomical scan and had scores on the MMSE and AVLT. Abbreviations: NADS, Nanjing Aging and Dementia Study; MCI, mild cognitive impairment; CARE, characterizing AD risk event; EM, episodic memory; IPS, information processing speed; VF, visuospatial function; MMSE, Mini-Mental State Examination. A statistical threshold was set at a *p* < 0.05 (false discovery rate [FDR]-corrected]. Δ represents the changes.

The changes in CARE index score measured at baseline and the three-year follow-up were negatively correlated with changes in mini-mental state examination (MMSE) scores, and the composite Z scores of episodic memory, information processing speed, and visuospatial function in MCI (p < 0.05). (See Figure 5 and Supplementary Table 3.)

## DISCUSSION

The most novel result of this study is that the CARE index allows us to accurately predict MCI-to-AD progression on an individual subject basis across different cohorts.

This study showed no differences in the conversion rates in individuals with MCI after a 3-year follow-up between ADNI (26.1%) and NADS (28.6%), which agrees with these findings based on ADNI that showed the conversion rates of 26 to 48% after a 3-year follow-up (Devanand DP et al. 2008; Okello A et al. 2009). This suggests our results are unbiased and convincing.

Recently, an excellent study by Young and colleagues used an EBP model to develop a biomarker prognostic model that predicts the MCI-to-AD conversion over three years [17]. They obtained the relatively high balanced accuracy of 77%, sensitivity of 86%, and specificity of 69%. On the basis of their study, we have made significant improvements from a practical perspective, as follow: First, the CARE index only integrates widely available, cost-effective, and noninvasive markers (i.e., behavioral, structural, and functional MRI markers). In particular, we added brain functional connectivity, which improved the prediction performance with regard to MCI-to-AD progression. It is well established that the measures of brain connectivity at rest are sensitive to AD progression [18] and the incorporation of brain connectivity measures with other biomarkers can add further predictive information to the prognostic model [19, 20]. Second, the CARE index can be applied to predict MCI-to-AD progression in an independent population with a very high prediction performance.

A strength of this work is that the overall predictive performance of the CARE index was not only high but also has a fairly balanced sensitivity/specificity, which has significantly outperformed other existing work thus far. Several studies have developed models based on various combinations of CF, MRI, PET, and CSF biomarkers. They have applied these models to distinguish MCI converters from MCI non-converters over a three-year follow-up period, only attaining accuracies in the range of 0.49–0.75 and AUCs in the range of 0.64–0.76 [4–6, 10, 21]. The high prediction performance for the CARE index may be the reason why all selected markers are well-studied and well-established AD markers, These markers are significantly associated with conversion from MCI to AD [22, 23]. The structural and functional impairment of the hippocampus, hallmarks of AD, is consistently considered as a valuable predictor of progression from MCI to AD [24]. The default mode network (PCC as a core hub) abnormalities are associated with episodic memory impairment early in AD [25, 26], representing AD disease progression [27]. Furthermore, the CARE index is a staging system for disease monitoring, which can reveal a detailed evaluation of patient state. Using a baseline MRI marker, Wee et al. can predict the MCI-to-AD conversion with relatively high accuracy (AUC = 0.84) [13], although they report limited sensitivity (75.1%) and low specificity (63.5%). Furthermore, there are two studies that report results comparable to those in our study. Using CSF and plasma markers, Lehallier et al. report a good ability to predict the MCI-to-AD conversion within three years with relatively high accuracy (80%), sensitivity (88%), specificity (70%), and balanced accuracy (79%) [28]. However, they evaluate the predictive performance using a form of internal validation in the same dataset. This method may lead to overfitting in the predictive model [12, 29]. Using CF and MRI biomarkers, a recent study also achieved a relatively high accuracy (79.9%), sensitivity (83.4%), specificity (76.4%), AUC of 0.87, and balanced accuracy (79.9%) [30]*. However, they use* a nested stratified cross-validation procedure to evaluate the predictive performance in the same dataset [12, 29]. Using MRI and plasma biomarkers, Liu and colleagues achieved a high accuracy (96%), AUC of 0.82, and sensitivity (95%), but at the cost of a very low specificity (65%) [31]. Using MRI and functional MRI (functional connectivity) biomarkers, Serra and colleagues can predict MCI-to-AD progression with high accuracy (89.7%), sensitivity (84.6%), and specificity (93.8%) [19]. However, they performed a simple discriminant analysis with no form of internal or external validation. This may especially lead to overfitting in the predictive model. Furthermore, they used a relatively small sample size (a total of 31 MCI subjects) and a relatively short-term, two-year follow-up with a high conversion rate of 45.2% (14 converters from a total of 31 MCI subjects). This conversion rate after a two-year follow-up is considerably higher than the 35% and 37.5% reported by other studies [32, 33], respectively. A previous study indicated disease progression may present more dynamic features within two years and demonstrated relatively stable performance over two years [34]. It is of crucial importance to note that predicting the conversion over a short timespan means a proportion of the MCI subjects will likely convert to AD at a later time, which reduces the positive predictive value of the classification result [35]. Most importantly, the prediction performance of the models in all of the above-mentioned studies has not been validated in an independent cohort.

Another principal novelty of this study is that we were able to replicate our results in an independent cohort, which suggests that the CARE index is a robust and accurate model with good generalizability. Recently, a few studies have begun to address the generalization bottleneck; however, their prediction performance could not be transferred from one cohort to other cohorts [6, 15]. And they obtained a low robustness across cohorts, with low accuracies. The methodological differences may account for the CARE index’s superior prediction performance, robustness, and generalizability compared to those of their models. First, unlike above-mentioned models, the CARE index estimates the probabilities of occurrence and nonoccurrence of each biomarker on an individual basis by using the EBP model, rather than by dichotomizing biomarker status based on the cutoff point threshold or clinical diagnosis information [16]. The CARE index emphasizes the temporally dependent process of diverse pathophysiological events underlying AD development and can accurately stage each individual across the whole AD spectrum [16]. The probabilities of occurrence and nonoccurrence of each pathophysiological event are affected only by the disease progression of the individual and not by the whole nature of the different independent cohorts, such as multicenter or single-center, or cohort heterogeneity. Second, Prestia and colleagues’ models use high-dimensional input in prediction, which are often related to the so-called “curse of dimensionality” [36]. which significantly hampers modeling generalization. The explanation may be that many machine learning methods may lose their ability to generalize to unseen examples with the increase in the number of available input features, due to the discordance between the sample size and the increased dimensionality [4]. Therefore, the CARE index exhibits a remarkable advantage in predicting the MCI-to-AD conversion across different cohorts.

In the current study, our findings support the fact that combining different biomarkers can predict the conversion status and cognitive decline. The CARE index associated with conversion likely reflects disease severity, i.e., how close an individual is to a significant clinical transition. The CARE index integrates biomarkers derived from behavior, structural, and resting-sate functional MRI modalities. It has important clinical advantages such as cost-efficiency, noninvasiveness, very high test–retest reliability, strong validity for AD pathological process, and clinical popularity. Most importantly, the excellent predictive performance of CARE index in a multicenter study (ADNI) can be applied in a clinical single-center study (NADS). Therefore, the CARE index can be generalized for use in precisely selecting individuals with MCI for a clinical trial. It can aid the development of new disease-modifying drugs by assigning possible surrogate markers of disease progression, and it can reduce the number of subjects needed to detect a significant drug effect [37]. Ultimately, it is reasonable to speculate that it would be desirable for the CARE index to be available to clinicians.

Some limitations of this study deserve comment. First, sample sizes of P-MCI and N-MCI subjects in the separate ADNI and NADS cohorts, especially the P-MCI subjects, are relatively small. However, it is important to note that our supplementary analysis combining ADNI and NADS cohorts still showed a very high power in predicting conversion from MCI to AD. Therefore, our results remain convincing. Second, we only used parts of MCI subjects from ADNI cohorts due to our entry criteria, which may affect our results. Finally, this study assessed only the predictive power of the CARE index during a three-year follow-up period. Future studies with longer and multiple follow-up times (at four years, five years, and so on) are needed to refine and generalize our CARE index estimates. Furthermore, the diffusion tensor imaging (DTI) has been considered as an advanced technology on studying the MCI/AD patients. Therefore, the further studies will corroborate and extend high prediction performance of the CARE index and explore whether combining DTI, brain structural and functional MRI can improve this high prediction performance. Finally, other cognitive measures and non-invasive biomarkers may play crucial roles in improving prediction performance that were not addressed here (such as the CDR–sum of boxes and diffusion tensor imaging measures)

In conclusions, the CARE index is sufficiently robust and generalizable for predicting which MCI individuals, across datasets, will develop AD over three years. It can be usefully applied to select individual subjects with MCI for clinical trials and to predict with high sensitivity and specificity for early treatment which individual subjects with MCI will convert to AD in the future.

## METHODS

### Subjects

The data for study subjects were obtained from two independent datasets: ADNI and NADS.

### ADNI

Baseline data used in this study were consistent with data in our previously published study [16]. The details of the ADNI information are provided in SI Methods S.1.

We selected a total of 74 MCI subjects with a baseline diagnosis of amnestic MCI, based on the requirements in our previously published study [16]. Finally, 46 subjects had a three-year follow-up clinical diagnosis of MCI. According to the follow-up clinical diagnosis by the National Institute of Neurological and Communicative Disorders and Stroke or the Alzheimer’s Disease and Related Disorders Association (NINCDS-ADRDA) criteria for the diagnosis of probable AD [38], those MCI subjects who progressed to AD within 36 months of entering the study were labeled as progressive MCI (P-MCI), and those who did not progress were labeled as non-progressive MCI (N-MCI) subjects. The clinical statuses for P-MCI and N-MCI subjects are employed as the “ground truth” in our classification experiments as described below. The characteristics of the MCI subjects are provided in Table 1.

### NADS

The NADS study recruited 87 subjects with a baseline diagnosis of amnestic MCI status. Written informed consent was obtained from all of the participants, and the study was approved by the responsible Human Participants Ethics Committee of the Affiliated ZhongDa Hospital, Southeast University, Nanjing, China.

All amnestic MCI subjects met the diagnostic criteria proposed by Petersen and colleagues [39] and the current revised consensus criteria of the International Working Group on amnestic MCI [40]. The inclusion and exclusion criteria (see details in SI Methods S.2) used to choose subjects can be found in our previously published studies [41]. Finally, 56 subjects had a three-year follow-up clinical diagnosis of amnestic MCI. The clinical status of each MCI subject was reevaluated at 36 months and classified into the N-MCI and P-MCI groups, as described above. The characteristics of the MCI subjects are provided in Table 1.

### Neuropsychological assessment

In the NADS dataset, all subjects underwent a standardized clinical interview and comprehensive neuropsychological assessments that were performed by neuropsychologists (Dr. Gu, Gao, and Yan). These assessments included mini-mental state examination (MMSE), Mattis Dementia Rating Scale (MDRS); Auditory Verbal Learning Test–immediate recall (AVLT-IR); Auditory Verbal Learning Test–5-min delayed recall (AVLT-5-min-DR); Auditory Verbal Learning Test–20-min delayed recall (AVLT-20-min-DR); Logical Memory Test–immediate recall (LMT-IR); Logical Memory Test–20-min delayed recall (LMT-20-min-DR); Rey-Osterrieth Complex Figure Test (ROCFT); Rey-Osterrieth Complex Figure Test –20-min delayed recall (ROCFT-20min-DR); Trail-Making Tests A and B (TMT–A and B); Digital Symbol Substitution Test (DSST); Digit Span Test (DST); Stroop Color and Word Test A, B, and C; Verbal Fluency Test (VFT); Semantic Similarity (Similarity) test; and Clock Drawing Test (CDT). These tests were used to evaluate multi-domains of cognitive function, including general cognitive function, episodic memory, information processing speed, executive function, and visuo-spatial function, respectively. Note that all subjects in the NADS dataset had both scores of comprehensive neuropsychological assessment and at least one R-fMRI scan with corresponding anatomical scans at 3-year follow-up to investigate the links between the changes of characterizing AD risk event and the changes of neuropsychological performance. Furthermore, we have standardized the tests in the NADS dataset into the ADNI dataset when the cognitive measure scores obtained across different study datasets may have different testing composition.

### MRI data acquisition

### *ADNI dataset*

The ADNI data acquisition process is described at http://adni.loni.ucla.edu/. The details regarding image acquisition are provided in SI Methods S.4.

### *NADS dataset*

The details regarding image acquisition parameters are provided in SI Methods S.4 and in our previously published studies [42].

### Image preprocessing

Conventional preprocessing steps were conducted using Analysis of Functional NeuroImages (AFNI) software, SPM8, and MATLAB (Chen et al., 2016). The preprocessing allows for T1-equilibration (removing the first 15 s of R-fMRI data); slice-acquisition-dependent time shift correction (3dTshift); motion correction (3dvolreg); detrending (3dDetrend); despiking (3dDespike); spatial normalization (original space to the Montreal Neurological Institute [MNI] space, SPM8); averaging white matter and CSF signal retrieval (3dROIstats) using standard SPM white matter and CSF mask in the MNI space; white matter, CSF signal, and motion effect removal (3dDeconvolve); global signal removal necessity check (the global signal will be removed if necessary) [5]; and low-frequency band-pass filtering (3dFourier, 0.015–0.1Hz).

### Biomarker events, expected stage, and missing biomarker

According to our previously published studies [16], 10 well-studied AD biomarkers were selected, each representing an event that dynamically occurs along with AD progression. These biomarkers include three functional connectivity indices (FCI) from the hippocampus (HIP^FCI^), the posterior cingulate cortex (PCC^FCI^), and the fusiform gyrus (FUS^FCI^); two gray matter concentration indices (GMI) from the hippocampus (HIP^GMI^) and fusiform gyrus (FUS^GMI^); two CSF biomarkers (Aβ_1-42_ and p-tau levels); and three cognitive biomarkers (MMSE, ADAS-Cog, and AVLT scores). Detailed methods to extract FCI and GMI are provided in SI Methods S.6 and S.7.

The optimal temporal sequence, S^optimal^, is determined by the event-based probabilistic (EBP) model. The S^optimal^ sequence, in which these biomarker events occur, was well studied in our previously published work [16]. The mathematical detail of the EBP model is described previously [16, 17] and in SI Methods S.8, S.9, and S.10.

In the case of missing biomarkers, the mathematical detail of the S^optimal^ sequence of biomarker events with missing data is described in SI Methods S.8–S.12.

### Individual CARE index

We numbered each of the 10 biomarker events by their order of occurrence in S^optimal^; collectively, these events comprise the index for CARE index. Each subject’s CARE index score is defined as that at which the order number had the highest likelihood value in S^optimal^ (SI Methods S.11). Note that in this study, three biomarkers (two CSF biomarkers of Aβ1-42 and p-tau levels and ADAS-Cog scores) were missing in the NADS dataset. Therefore, the same biomarkers in the ADNI dataset were selected to be consistent with those in the NADS dataset to compute the CARE index.

### Statistical analysis

### *Demographic and neuropsychological data*

The statistical analyses were conducted with SPSS 22.0 software. The two-sample *t*-test, chi-square (χ^2^) test, and Mann-Whitney *U* tests were used to compare the differences in demographic data, neuropsychological performance, each individual biomarker feature, and the CARE index between N-MCI and P-MCI subjects. A statistical threshold was set at a *p* < 0.05.

### *MCI conversion prediction*

We evaluated the power of the CARE index and of individual biomarkers to discriminate P-MCI subjects from N-MCI subjects with the use of receiver operating characteristic (ROC) curves [43]. To demonstrate the CARE index’s power to predict clinical progression from MCI to AD, the area under the ROC (AUC) values were employed to compare the CARE index and individual biomarkers using a nonparametric method for correlated samples [44]. The optimal cutoff value of the CARE index for discriminating P-MCI subjects from N-MCI subjects was extracted from the ADNI dataset, generating optimal sensitivity and specificity values, accuracy, odds ratio (OR), and relative risk (RR). Furthermore, we applied the CARE index classifier obtained from the ADNI dataset to the NADS dataset to validate the generalizability of the classifier in discriminating between P-MCI and N-MCI subjects.

To compare the stability and generalizability of the CARE index to discriminate between P-MCI and N-MCI subjects with those of individual biomarkers, we also applied the optimal classifier of each biomarker obtained from the ADNI dataset to the NADS dataset. To avoid unbalanced class frequency to lead to discrepancies between sensitivity and specificity [7, 35], we also reported the balanced accuracy: defined as (sensitivity + specificity)/2 [45]. To directly observe the power of each biomarker in distinguishing P-MCI subjects from N-MCI subjects across datasets, we ranked the AUC, optimal sensitivity and specificity, accuracy, and balanced accuracy of the CARE index and individual biomarkers.

In addition, to avoid limitations due to the relatively small sample size and the differences in the predictive power of the CARE index between the ADNI and NADS cohorts due to the differences of MCI heterogeneity in general, we performed a supplementary analysis with the combined cohort of ADNI and NADS datasets.

### *Behavioral significance of the changes in CARE index measured at baseline and the three-year follow-up*

We performed a multiple linear regression model analysis to examine the relationships between the changes in CARE index and the changes in cognitive performance or clinical variables at baseline and the three-year follow-up in MCI subjects (See details in *SI Methods S.13*). The statistical threshold was set at a *p* < 0.05 (FDR-corrected).

## Supplementary Material

Supplementary Information
