# Supplementary material for: Predicting progression from mild cognitive impairment to Alzheimer’s disease on an individual subject basis by applying the CARE index across different independent cohorts
Source: Aging (Albany NY). 2019 Apr 30;11(8):2185–201. doi: 10.18632/aging.101883 (PMC6520016; doi:10.18632/aging.101883)
Supplement: Supplementary Information [file aging-11-101883-s001.pdf]

Supplementary Methods

Participants for the ADNI dataset

Data used in this study were obtained from the Alzheimer's Disease Neuroimaging Initiative (ADNI) database (http://adni.loni.usc.edu). ADNI was launched in 2003 by the National Institute on Aging (NIA), the National Institute of Biomedical Imaging and Bioengineering (NIBIB), the US Food and Drug Administration (FDA) [1], private pharmaceutical companies, and nonprofit organizations, as a $60 million, five-year public-private partnership. ADNI is an observational study with both cross-sectional and longitudinal follow-up components. The primary goal of ADNI has been to test whether neuroimaging, fluid and genetic biomarkers, and cognitive assessments can be combined to measure the progression of mild cognitive impairment (MCI) and early Alzheimer's disease (AD).

This study employed data from the ADNI 2, which includes 225 subjects with three clinical groups (cognitively normal [CN], MCI, and AD) (https://adni.loni._usc.edu/wp-content/uploads/2008/07/adni2-procedures-manual.pdf downloaded May 19, 2016). All subjects and their study partners completed the informed consent process, and the study protocols were reviewed and approved by the Institutional Review Board at each ADNI data collection site.

The general eligibility, inclusion, and exclusion criteria for ADNI subjects can be found on the ADNI website (www.adni-info.org). We selected a total of 74 MCI subjects with a baseline diagnosis of amnestic MCI, based on the following requirements: First, all subjects had at least one resting-state functional magnetic resonance imaging (R-fMRI) scan with corresponding anatomical scans. Second, all subjects had cerebrospinal fluid (CSF), amyloid beta (Aβ), and phosphorylated tau (p-tau) concentration values. Third, all subjects had scores on the Mini-Mental State Examination (MMSE), modified 13-item Alzheimer's Disease Assessment Scale-Cognitive Subscale (ADAS-Cog), and Rey Auditory Verbal Learning Test (AVLT) (immediate recall score, i.e., the sum of trials 1 to 5). Of note, in this study, the CSF, p-tau, and ADAS-Cog biomarkers were used only to estimate the optimal temporal sequence of events by using event-based probabilistic model. Finally, in this study, 46 subjects had a three-year follow-up clinical diagnosis of MCI and met criteria for inclusion as part of either a nonprogressive MCI (N-MCI) group or a progressive MCI (P-MCI) group, depending on whether they progressed to AD-type dementia at the three-year follow-up. At study entry (baseline), all subjects underwent a standardized clinical interview, cognitive/functional assessments, structural MRI, and R-fMRI scans. The clinical status of each MCI subject was reevaluated at the 36-month follow-up time point. According to the follow-up clinical diagnosis by the NINCDS-ADRDA criteria for the diagnosis of probable AD [2], those MCI subjects who progressed to AD-type dementia within 36 months of entering the study were labeled as P-MCI, those who did not progress were labeled as N-MCI subjects. The clinical status for N-MCI and P-MCI subjects is employed as the "ground truth" in our classification experiments, as described below. Note that most of the subjects included in the ADNI dataset had not met both comprehensive neuropsychological assessment scores and had at least one R-fMRI scan with corresponding anatomical scans at the three-year follow-up. Therefore, data from the ADNI dataset were not used to investigate the links between the changes of the characterizing AD risk events and the changes of neuropsychological performance.

There are 12 P-MCI subjects who progressed to AD and 34 N-MCI subjects who did not within 36 months of entering the study. Among the 34 N-MCI subjects, four MCI subjects reverted to normal cognitive status and remained dementia-free, the other 30 MCI subjects remained cognitively stable.

Participants for the NADS dataset

The NADS study recruited 87 subjects with a baseline diagnosis of amnestic MCI status, through normal community health screening, newspaper advertisement, and hospital outpatient service. Written informed consent was obtained from all of the participants, and the study was approved by the responsible Human Participants Ethics Committee of the Affiliated ZhongDa Hospital, Southeast University, Nanjing, China. To be considered for inclusion, participants had to have had functional and structural MRIs performed in ZhongDa Hospital, which is affiliated with Southeast University, on a Neuroscience Imaging Center 3T scanner.

All amnestic MCI subjects met the diagnostic criteria proposed by Petersen and colleagues [3] and the revised consensus criteria of the International Working Group on amnestic MCI [4], including the following: (1) subjective memory impairment was corroborated by the subject and an informant, (2) objective memory performance was documented according to an Auditory Verbal Learning Test-delayed recall score that was within ≤ 1.5 SD of age- and education-adjusted norms (the cutoff was ≤ 4 correct responses on 12 items for ≥ 8 years of education), (3) normal general cognitive function was evaluated by a MMSE of 24 or higher, (4)

a Clinical Dementia Rating of 0.5 with at least a 0.5 rating in the memory domain, (5) no or minimal impairment in daily living activities, and (6) the absence of dementia, or symptoms that were sufficient to meet the National Institute of Neurological and Communicative Disorders and Stroke or the AD and Related Disorders Association criteria for AD. The exclusion criteria were as follows: (1) a past history of known stroke (modified Hachinski score of > 4), alcoholism, head injury, Parkinson's disease, epilepsy, major depression (excluded using a Self-Rating Depression Scale), or other neurological or psychiatric illness (excluded by clinical assessment and case history), (2) major medical illness (e.g., cancer, anemia, or thyroid dysfunction), (3) severe visual or hearing loss, and (4) a T2-weighted MRI showing major white matter (WM) changes, infarction, or other lesions (two experienced radiologists analyzed the scans). Finally, 56 subjects had a three-year follow-up clinical diagnosis of amnestic MCI.

The clinical status of each MCI subject was reevaluated at 36 months and classified into the N-MCI and P-MCI groups, as described above. There were 16 P-MCI and 40 N-MCI subjects in the NADS study. Of 40 N-MCI subjects, six MCI subjects reverted to normal cognitive status and remained dementia-free; the other 34 MCI subjects remained cognitively stable.

Neuropsychological assessments for the NADS dataset

All subjects underwent a standardized clinical interview and comprehensive neuropsychological assessments that were performed by neuropsychologists, including MMSE, Mattis Dementia Rating Scale (MDRS); Auditory Verbal Learning Test–immediate recall (AVLT-IR); Auditory Verbal Learning Test–5-min delayed recall (AVLT-5-min-DR); Auditory Verbal Learning Test–20-min delayed recall (AVLT-20-min-DR); Logical Memory Test–immediate recall (LMT-IR); Logical Memory Test–20-min delayed recall (LMT-20-min-DR); Rey-Osterrieth Complex Figure Test (ROCFT); Rey-Osterrieth Complex Figure Test – 20-min delayed recall (ROCFT-20min-DR); Trail-Making Tests A and B (TMT–A and B); Digital Symbol Substitution Test (DSST); Digit Span Test (DST); Stroop Color and Word Test A, B, and C; Verbal Fluency Test (VFT); Semantic Similarity (Similarity) test; and Clock Drawing Test (CDT). These tests were used to evaluate general cognitive function, episodic memory, information processing speed, executive function, and visuo-spatial function.

Image acquisition for the ADNI dataset and the NADS dataset

The ADNI data acquisition process is described at http://adni.loni.ucla.edu/. Briefly, R-fMRI datasets were scanned on 3.0 Tesla MRI scanners (Philips, Netherlands). Axial R-fMRI images of the whole brain were obtained in seven minutes with a single-shot gradient echo planar imaging (EPI) sequence. High-resolution MP-RAGE (magnetization-prepared rapid gradient-echo) 3-D sagittal T1-weighted images also were acquired.

MRI images for the NADS dataset were acquired using a 3.0 Tesla Verio Siemens scanner (Erlangen, Germany) with a 12-channel head-coil at ZhongDa Hospital, which is affiliated with Southeast University. Resting-state functional images including 240 volumes were obtained using a gradient-recalled echo-planar imaging (GRE-EPI) sequence, with repetition time (TR) = 2000 ms, echo time (TE) = 25 ms, flip angle (FA) = 90°, acquisition matrix = 64 × 64, field of view (FOV) = 240 mm × 240 mm, thickness = 4.0 mm, gap = 0 mm, number of slices = 36, and voxel size = 3.75 × 3.75 × 4 mm^3. High-resolution T1-weighted axial images covering the whole brain were acquired by 3D magnetization prepared rapid gradient echo (MPRAGE) sequence as described below: TR = 1900 ms, TE = 2.48 ms; FA = 9°, acquisition matrix = 256 × 256, FOV = 250 × 250 mm, thickness = 1.0 mm, gap = 0 mm, number of slices = 176, and voxel size = 1 × 1 × 1 mm^3. Additionally, routine axial T2-weighted images were acquired to rule out subjects with major WM changes, cerebral infarction or other lesions using flair sequence as below: TR = 8400 ms, TE = 94 ms, FA= 150°, acquisition matrix = 256 × 256, FOV = 230 ×2 30 mm, thickness = 5.0 mm, gap = 0 mm, and number of slices = 20.

Image preprocessing

Conventional preprocessing steps were conducted using Analysis of Functional NeuroImages (AFNI) software, SPM8, and MATLAB. The preprocessing allows for T1-equilibration (removing the first 15 s of R-fMRI data); slice-acquisition-dependent time shift correction (3dTshift); motion correction (3dvolreg); detrending (3dDetrend); despiking (3dDespike); spatial normalization (original space to the Montreal Neurological Institute [MNI] space, SPM8); averaging white matter and CSF signal retrieval (3dROIstats) using standard SPM white matter and CSF mask in the MNI space; white matter, CSF signal, and motion effect removal (3dDeconvolve); global signal removal necessity check (the global signal will be removed if

necessary) [5]; and low-frequency band-pass filtering (3dFourier, 0.015–0.1Hz).

Functional connectivity indices of the regions of interest

This study calculated the functional connectivity indices (FCI) of the three regions of interest (ROIs): bilateral hippocampus (HIP^{FCI}), posterior cingulate cortex (PCC^{FCI}), and fusiform gyrus (FUS^{FCI}). The details regarding the FCI calculation stream were found in our previously published study [6]. First, the whole cerebral cortex was separated into 90 regions based on the Automated Anatomical Labeling (AAL) template, and the blood oxygen level dependent (BOLD) time series of each region was extracted using the AAL template mask from the preprocessed resting-state dataset [7]. Second, functional connectivity between each ROI and the other brain regions was calculated using the Pearson cross-correlation analysis. Thus, a vector consisting of 89 cross-correlation coefficient (CC) values for each ROI was obtained. Finally, each ROI's FCI value—identified separately as HIP^{FCI}, PCC^{FCI}, and FUS^{FCI} was calculated by summating 89 CC values within each ROI's vector and averaging them across each pair of bilateral ROIs.

$i=k+1$

Gray matter index

For each subject, the gray matter index [8] of each brain region (using the same AAL template) was calculated using SPM8 software (www.fil.ion.ucl.ac.uk/spm/software/spm8/). First, the anatomical image of each individual's brain was normalized into the Montreal Neurological Institute (MNI) space. Second, the gray matter of the whole brain was segmented and separated from WM and CSF areas, and a threshold of 0.8 was used to exclude non-gray-matter areas. Third, each region's gray matter concentration index was determined by summing the gray matter concentration values of all voxels within the region and averaging across each pair of bilateral ROIs.

Event-based probabilistic model

Given that a set of N events, $E_1, E_2, \ldots, E_N$, is measured by N biomarkers, $x_1, x_2, \ldots, x_N$, respectively, the temporal order of events, S={s(1), s(2), …, s(N)}, is calculated by a permutation of the integers 1, …, N. For subjects j=1, …, J, the dataset X could be regarded as $X=\{X_1, X_2, \ldots, X_J\}$. Specifically, X_J represents the subject j data that is given by $X_j=\{x_{1j}, x_{2j}, \ldots, x_{Nj}\}$, where x_{ij} is the ith biomarker measurement for subject j. This study determined the optimal temporal order in a data-driven manner, based on the criteria that the optimal temporal order, defined as the $S^{optimal}$, yielded the highest probability in measuring dataset X. That is, the p(X|S) value of the $S^{optimal}$ sequence was calculated to be maximal among all of the possible sequences. To accomplish this objective, we first estimated the likelihood of measurement x_{ij} given that biomarker event E_i has or has not occurred. These likelihoods are labeled below:

$p(x_{ij}|E_i)$ = likelihood of measurement x_{ij} given that event E_i has occurred (1)

$p(x_{ij}|\neg E_i)$ = likelihood of measurement x_{ij} given that event E_i has *not* occurred (2)

We assumed that subject j is at stage k, although the authentic biomarkers sequence and the subject's stage were unavailable. This means that for subject j, events Es(1), Es(2), …, Es(k) already have occurred, and eevents $E_{s(k+1)}$, $E_{s(k+2)}$, …, $E_{s(N)}$ have not occurred. The likelihood of data X_j given the sequence S and the subject's stage at k was obtained using the formula below:

$$p(X_j|S,k) = \prod_{i=1}^{k} p(x_{ij}|E_{s(i)}) \prod_{i=k+1}^{N} p(x_{ij}|\neg E_{s(i)}) \quad (3)$$

Where $\prod_{i=1}^{k} p(x_{ij}|E_{s(i)})$ is the overall likelihood of measurements given that corresponding events have already occurred, $\prod_{i=k+1}^{N} p(x_{ij}|\neg E_{s(i)})$ the overall likelihood of measurements given that these events have not yet occurred. Then, we obtained the likelihood of data X_j in the condition of sequence S by summing the likelihood values of data X_j across all possible stages within sequence S, as shown in Equ. (4) below:

$$p(X_j|S) = \sum_{k=0}^{N} p(k) p(X_j|S,k) \quad (4)$$

Next, we combined the measurements of all subjects, j=1, …, J, assuming that the intersubject relationship is independent:

$$p(X|S) = \prod_{j=1}^{J} p(X_j|S) = \prod_{j=1}^{J} \sum_{k=0}^{N} p(k) p(X_j|S,k)$$
$$= \prod_{j=1}^{J} \sum_{k=0}^{N} p(k) [\prod_{i=1}^{k} p(x_{ij}|E_{s(i)}) \prod_{i=k+1}^{N} p(x_{ij}|\neg E_{s(i)})] \quad (5)$$

In theory, the above analysis needs to be repeated for each possible sequence to determine the sequence $S^{optimal}$ with the maximal value of $p(X|S)$. However, such a computation strategy is extremely time consuming; total calculation times in this study would be 2.7942e+009, given that there are 10 biomarker events, 11 possible stages (including stage 0), and 70 subjects (cognitively normal [CN] and AD groups only) involved. Therefore, we employed a greedy algorithm to improve processing efficiency.

Table S1. The order of the 10 progressive events in AD development represented by the 10 well-studied biomarkers.

E1	E2	E3	E4	E5	E6	E7	E8	E9	E10
HIP^{FCI}	PCC^{FCI}	Aβ1-42	p-tau	MMSE	ADAS-Cog	AVLT	HIP^{GMI}	FUS^{GMI}	FUS^{FCI}

Event occurrence and nonoccurrence distribution modeling

We used a mixture model of two Gaussian distributions to fit the event data from the CN and AD groups, based on the assumption that an event occurring and an event not occurring are estimated by a mixed distribution of normal and abnormal groups. The fitted Gaussian distributions separated the data into two groups, i.e., abnormal (event occurred) and normal (event did not occur), similar to the approach by Young et al. [9]. Notably, we modified Young et al.'s approach by applying a k-mean clustering algorithm to separate the whole distribution into two clusters before applying the Gaussian mixture model fitting. This modified modeling method led to high consistency in the obtained model.

Self-growing greedy algorithm

The amount of time such an analysis would take to find a global optimal result is unpredictable due to the randomized initial sequence, and it may be quite long due to the inevitable searching loop. Therefore, we developed a new greedy algorithm to address this deficiency. The greedy algorithm explores the globally optimal solution by making the locally optimal choice at each stage, in a greedy heuristic manner. The greedy Markov chain Monte Carlo (MCMC) algorithm is a useful approach to find globally optimal results. Specifically, we started with a set of all possible initial root sequences, each of which consisted of two randomly selected events from the 10 biomarker events total. Second, for each initial sequence S, we generated the children of S by inserting a randomly selected event from the remaining events. Third, we selected the children sequence with the maximal $p(X|S)$ value; this replaced the initial sequence. Then, we entered another randomly selected event into the sequence and repeated the second and the third steps until no events were left. Thus, we generated whole sequences for each root sequence. Ultimately, we determined the sequence with the maximum $p(X|S)$ value as the final optimal sequence, $S^{optimal}$. We repeated this greedy algorithm 100 times to ensure the $S^{optimal}$ had a high reliability. This study used 45 CN and 25 AD subjects to determine the $S^{optimal}$. Note that the MCI subjects were not used to train the $S^{optimal}$.

The $S^{optimal}$ reflects the order in which the sequential pathophysiological events occurred and provides a numeric score to measure disease progression from one stage to the next.

CARE index score calculation based on the obtained sequence

Using the following equation to determine each subject's CARE index score, we calculated the likelihood value of *k* at each possible stage in the sequence and defined the CARE index score as that at which *k* had the highest likelihood value at the $S^{optimal}$:

$$argmax_k P(k) = \prod_{i=1}^{k} p\left(x_{ij} \middle| E_{S^{optimal}(i)}\right) \prod_{i=k+1}^{N} p\left(x_{ij} \middle| \neg E_{S^{optimal}(i)}\right) \quad (6)$$

In Equ. 6, implications of $\prod_{i=1}^{k} p\left(x_{ij} \middle| E_{S^{optimal}(i)}\right)$ and $\prod_{i=k+1}^{N} p\left(x_{ij} \middle| \neg E_{S^{optimal}(i)}\right)$efer to those in Equ. 3, except that the optimal sequence, $S^{optimal}$, is obtained.

Mathematical detail of missing biomarkers

Biomarker events

Ten well-studied AD biomarkers, as described above, were selected (Table S1); each represents an event that occurs along with AD progression.

Weighted average stage

The mathematical detail of event-based probabilistic (EBP) model is described earlier [6]. The EBP model determines the optimal order of biomarker events (i.e., $S^{optimal} = \{E1, E2, E3, E4, E5, E6, E7, E8, E9, E10\}$). It also determines the likelihood of subject j being in stage k, given the biomarker measurement X_j and $S^{optimal}$, by the formula below:

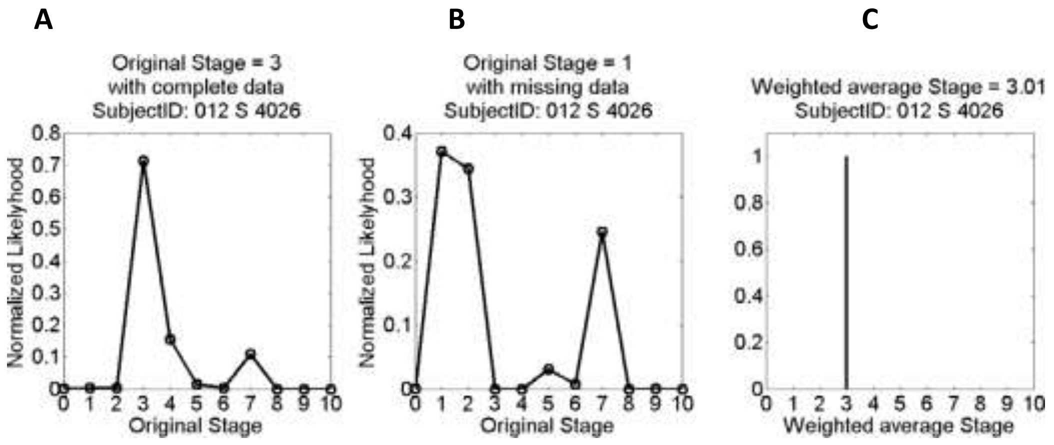

Figure S1. Advantage of the weighted average stage calculation. **(A)** Th subject's stage is 3 with complete data and the original method. **(B)** The subject's stage is 1 with missing data and the original method. **(C)** The subject's WA stage is 3.01 calculated with the formula shown in the figure. The difference is only 0.01 compared with the original stage calculated with the complete data. Weighted average Stage = 0x0+1x0.37+2x0.34+3x0+4 x0+5x0.03+6x0.01+7x0.25+ 8x0+9x0+10x0 = 3.01

$$p(X_j|S,k) = \prod_{i=1}^{k} p(x_{ij}|E_{s(i)}) \prod_{i=k+1}^{N} p(x_{ij}|\neg E_{s(i)}) \quad (7)$$

where $X_j=\{x_{1j}, x_{2j}, \ldots, x_{Nj}\}$, and x_{ij} is the ith biomarker measurement for subject j. The normalized likelihood is defined as:

$$p_{norm}(X_j|S,k) = A_j * p(X_j|S,k), \quad (8)$$

where normalization factor A_j is determined by:

$$\sum_{k=0}^{N} p_{norm}(X_j|S,k) = A_j * \sum_{k=0}^{N} p(X_j|S,k) = 1 \quad (9)$$

The weighted average (WA) stage $k_{j,weighted\ average}$ for subject j is defined as

$$k_{j,weighted\ average} = \sum_{k=0}^{N} k * p_{norm}(X_j|S,k) \quad (10)$$

$S^{optimal}$ in missing biomarkers

In the previous EBP model [6], the individual subject's disease stage was determined by the "winner take all" approach, i.e., the stage *k* with the highest likelihood value determines the subject's disease stage. However, this poses a potential problem when a biomarker is missing. For example, when a subject's disease stage corresponds to the missing marker *k*, it is impossible to determine the subject's disease stage to be *k* in the "winner take all" approach. The disease stage will fall to the next available highest likelihood stage, most likely $k-1$ or $k+1$. To address this problem, here we employ the WA stage defined in Equ (10). Theoretically, the WA stage of a subject can be *k* even when the corresponding marker is missing; this is demonstrated with a representative subject in Fig. S1. In the case of missing biomarker $i_{missing}$, $p(x_{i_{missing}j}|E_{s(i_{missing})})$ and $p(x_{i_{missing}j}|\neg E_{s(i_{missing})})$ were set to be 1. This is equivalent to removing them from Equ. (7) without having to modify the existing programs. Meanwhile, for k=$i_{missing}$, $p(X_j|S,k)$ was set to 0. With this numerical modification, neither the analytical equations (7–10) nor the existing programs from [6] needed to be modified. However, for clarity, Equ. (7) was rewritten to account for missing biomarker(s):

$$p(X_j|S,k) = 0, \quad k = i_{missing}$$

$$p(X_j|S,k) = \prod_{i=1}^{k} p(x_{ij}|E_{s(i)}) \prod_{i=k+1}^{N} p(x_{ij}|\neg E_{s(i)}), i \neq i_{missing}$$

The program was employed to calculate $S^{optimal}$ when the biomarkers were missing. The WA stage for each subject was calculated.

A representative subject

By employing the WA calculation, theoretically, the WA stage of a subject can be *k* even when the corresponding marker is missing. This benefit is demonstrated with a representative subject. Fig. S1 shows the advantage of the WA stage calculation. Fig S1A shows that the subject's stage is 3 with complete data and the original method; Fig. S1B shows that the

subject's stage is 1 with missing data and the original method. Note: With missing data at stages 3 and 4, the normalized likelihood of stages 1, 2, 5, and 7 increases and is maximized at stage 1. Compared to that calculated using complete data (stage 3), the result with missing data (stage 1) is two stages lower; Fig. S1C shows that the subject's WA stage is 3.01 calculated with the formula shown in the figure. The difference is only 0.01 compared with the original stage calculated with the complete data.

Statistical analysis

Demographic and neuropsychological data

To increase statistical power by reducing random variability, this study composited the neuropsychological tests into four cognitive domains and transformed the raw scores into four composite Z scores, as previously described [10-12]. First, for each neuropsychological test, the individual raw scores were transformed to Z scores, according to the mean and standard deviation of the scores for all subjects. The following is the equation for Z transformation:

$$Z_i = \frac{r_i - \bar{r}}{S},$$

where Z_i is the Z score of the ith subject, r_i is the raw score of the ith subject, $\bar{r}$ is the average raw score of the neuropsychological test for all subjects, and S is the standard deviation of the scores. Notably, for tests measured in time, including TMT–A, TMT–B, Stroop A, Stroop B, and Stroop C, the raw scores were defined as the reciprocal of the time required for the test. Then, each cognitive domain's composite Z score was determined by averaging the Z scores related to the tests. We divided these tests into four cognitive domains: episodic memory (six tests, including AVLT–IR, AVLT–5-min-DR, AVLT–20-min-DR, LMT–IR, LMT–20-min-DR, and ROCFT–20-min-DR), information processing speed (four tests, including DSST, TMT-A, Stroop A, and Stroop B), visuospatial function (two tests, including CFT and CDT), and executive function (six tests, including VFT, DST, TMT-B, Stroop C, and Similarity). Bonferroni correction for multiple comparisons was performed at a significance level of $p < 0.0125$ ($p = 0.05/4$ composite scores).

Supplementary Tables

Table S1. Comparison of the conversion rates of different MCI state transitions between ADNI and NADS datasets.

Dataset	Total	MCI	
		N-MCI	P-MCI
ADNI	46	34(73.91%)	12(26.09%)
NADS	56	40(71.43%)	16(28.57%)
χ^2		0.078	
p		0.78	

Abbreviations: ADNI, Alzheimer's Disease Neuroimaging Initiative; NADS, Nanjing Aging and Dementia Study, MCI, mild cognitive impairment; AD, Alzheimer's Disease; P-MCI, progressive MCI, including MCI subjects who progressed to AD-type dementia at the three-year follow-up; N-MCI, nonprogressive MCI, including MCI subjects who did not progress to dementia at the three-year follow-up.

Table S2. Sensitivity for P-MCI, specificity for N-MCI, and AUC and accuracy of CARE index and each of seven selected biomarker indices in the ADNI, NADS, and combined datasets.

		AUC				Sensitivity		Specificity		Accuracy		Balanced Accuracy		
	Predictors	Rank	P-MCI + N-MCI (n=46)	95%CI	p	Rank	P-MCI (n=12)	Rank	N-MCI (n=34)	Rank	P-MCI + N-MCI (n=46,%)	**Rank**	**%**	**Opt. Threshold**
ADNI	CARE index	**1**	0.809	0.68-0.94	0.02	**2**	0.75	**1**	0.82	**1**	80.4	1	78.7	6.54
	MMSE	**6**	0.578	0.42-072	0.42	**2**	0.75	**8**	0.44	**2**	76.1	8	59.6	28.5
	AVLT	**3**	0.761	0.61-0.87	0.08	**6**	0.67	**2**	0.79	**8**	52.2	3	73.0	28.5
	HIP^{FCI}	**8**	0.515	0.36-0.67	0.88	**8**	0.50	**4**	0.71	**5**	65.2	7	60.3	0.66
	PCC^{FCI}	**5**	0.635	0.48-0.77	0.17	**1**	0.83	**6**	0.50	**6**	58.7	5	66.7	2.28
	FG^{FCI}	**4**	0.659	0.51-0.79	0.10	**6**	0.67	**4**	0.71	**4**	69.6	4	68.6	10.57
	HIP^{GMI}	**2**	0.762	0.61-0.88	0.01	**2**	0.75	**3**	0.74	**3**	73.9	2	74.3	0.40
	FG^{GMI}	**6**	0.578	0.42-0.72	0.42	**2**	0.75	**6**	0.50	**7**	56.5	6	62.5	0.55

		AUC				Sensitivity		Specificity		Accuracy		Balanced Accuracy		
NADS		Rank	P-MCI + N-MCI (n=56)	95%CI	p	Rank	P-MCI (n=16)	Rank	N-MCI (n=40)	Rank	P-MCI + N-MCI (n=56,%)	**Rank**	**%**	**Opt. Threshold**
	CARE index	**2**	**0.861**	0.74-0.94	0.00	**1**	0.81	**1**	0.90	**1**	87.5	1	85.6	6.87
	MMSE	**3**	**0.837**	0.71-0.92	0.00	**6**	0.63	**1**	0.90	**3**	82.1	3	76.3	25.50

	AVLT	**1**	**0.876**	0.74-0.95	0.00	**2**	0.75	**1**	0.90	**2**	83.9	2	82.5	27.26
	HIP^{FCI}	**6**	**0.588**	0.45-0.72	0.31	**2**	0.63	**8**	0.60	**8**	60.7	7	61.3	-0.3
	PCC^{FCI}	**7**	**0.567**	0.43-0.70	0.44	**4**	0.69	**7**	0.62	**6**	64.3	6	65.6	4.98
	FG^{FCI}	**8**	**0.563**	0.42-0.70	0.47	**8**	0.50	**6**	0.70	**6**	64.3	8	60.0	11.58
	HIP^{GMI}	**4**	**0.811**	0.68-0.90	0.00	**4**	0.69	**4**	0.82	**4**	78.6	4	75.6	0.43
	FG^{GMI}	**5**	**0.634**	0.50-0.76	0.12	**6**	0.63	**5**	0.72	**5**	67.9	5	66.3	0.56

			**AUC**			**Sensitivity**		**Specificity**			**Accuracy**	**Balanced Accuracy**		
		Rank	P-MCI + N-MCI (n=102)	95%CI	p	Rank	P-MCI (n=28)	Rank	N-MCI (n=74)	Rank	P-MCI + N-MCI (n=102,%)	**Rank**	**%**	**Opt. Threshold**
**ADNI+ NADS**	CARE index	**1**	**0.839**	0.75-0.90	0.00	**1**	0.79	**1**	0.85	**1**	83.3	1	82.0	6.57
	MMSE	**4**	**0.715**	0.62-0.80	0.00	**8**	0.57	**4**	0.64	**4**	62.8	5	62.1	26.50
	AVLT	**2**	**0.826**	0.74-0.90	0.00	**3**	0.71	**1**	0.85	**2**	81.4	2	78.3	28.26
	HIP^{FCI}	**7**	**0.563**	0.46-0.66	0.33	**6**	0.61	**6**	0.61	**7**	60.8	7	60.8	0.66
	PCC^{FCI}	**8**	**0.532**	0.43-0.63	0.62	**2**	0.75	**8**	0.39	**8**	49.0	8	59.1	2.30
	FG^{FCI}	**6**	**0.598**	0.50-0.69	0.13	**6**	0.61	**4**	0.64	**4**	62.8	5	62.1	11.72
	HIP^{GMI}	**3**	**0.779**	0.69-0.86	0.00	**3**	0.71	**3**	0.74	**3**	73.5	3	72.9	0.43
	FG^{GMI}	**5**	**0.601**	0.50-0.70	0.12	**5**	0.68	**7**	0.59	**6**	61.8	4	63.7	0.56

Abbreviations: ADNI, Alzheimer's Disease Neuroimaging Initiative; NADS, Nanjing Aging and Dementia Study; CARE, characterizing AD risk events; P-MCI, progressive MCI, including MCI subjects who progressed to AD-type dementia at the three-year follow-up; N-MCI, nonprogressive MCI, including MCI subjects who did not progress to dementia at the three-year follow-up; MCI, mild cognitive impairment; AD, Alzheimer's Disease; AVLT, Rey Auditory Verbal Learning Test; MMSE, Mini-Mental State Examination; HIP, hippocampus; PCC, posterior cingulate cortex; FUS, fusiform gyrus; GM, gray matter; GMI, gray matter indices; FCI, functional connectivity indices; Opt, optimal.

Table S3, related to Figure 4. The correlation between the changes in CARE index and changes in cognitive performance or clinical variables measured at baseline and 3-year follow-up in MCI subjects from NADS dataset.

Item	Statistic	The correlation between CARE index and cognitive domain and clinical variables in:				
		EM	EF	IPS	VF	MMSE
**NADS dataset**						
	r	-0.5136	0.0206	-0.4028	-0.3764	-0.3417
	p	0.0004*	0.8944	0.0067*	0.0118*	0.0232*

Abbreviations: MCI, mild cognitive impairment; CARE, characterizing AD risk events; EM, episodic memory; EF, executive function; IPS, information processing speed; VF, visuospatial function; MMSE, Mini Mental State Exam; NADS, Nanjing Aging and Dementia Study. A statistical threshold was set at a $p < 0.05$ (false discovery rate [FDR]-corrected). * $P_{\text{FDR-corrected}} < 0.05$.

Supplementary Figures

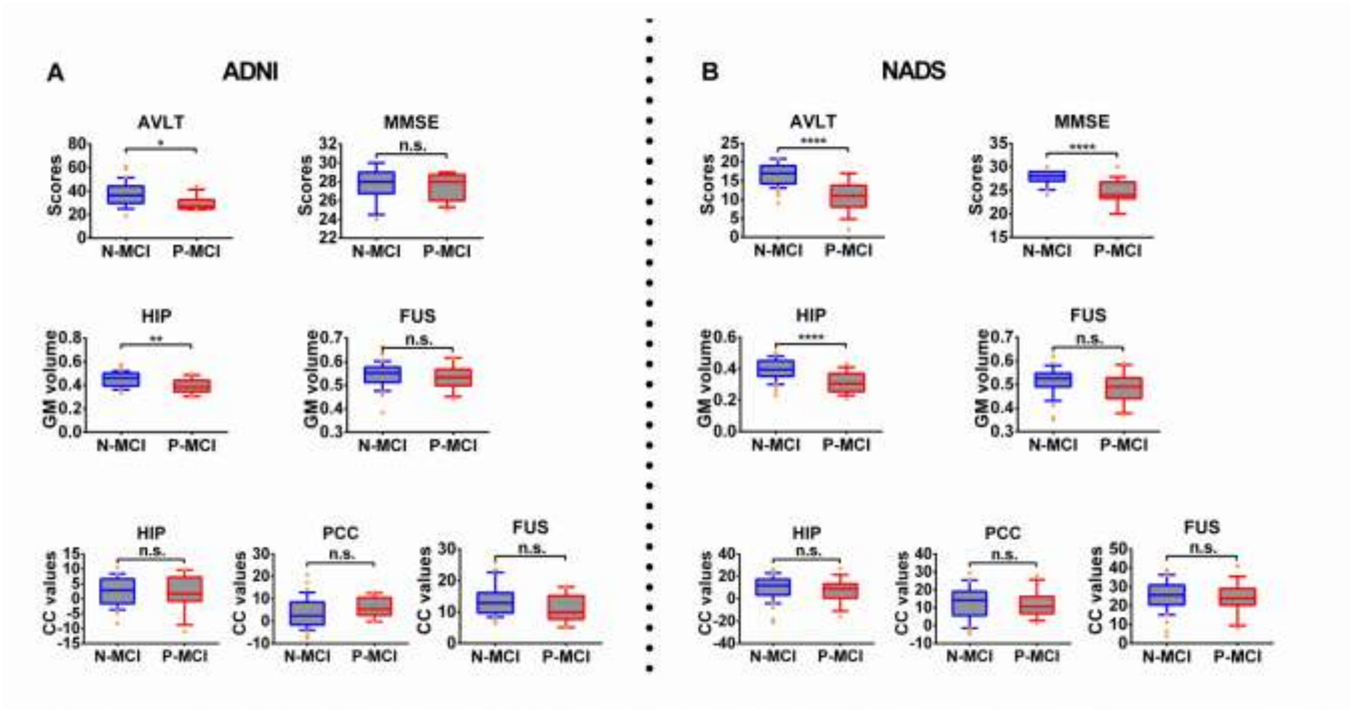

Figure S2, related to Figures 2 and 3. Scores of biomarkers at baseline from ADNI and NADS datasets. (**A**) Boxplot representing comparisons of index value of biomarkers in N-MCI and P-MCI at baseline from ADNI dataset. (**B**) Boxplot representing comparisons of index value of biomarkers in N-MCI and P-MCI at baseline from NADS dataset, * $p < 0.05$, ** $p < 0.01$, *** $p < 0.001$, **** $p < 0.0001$, n.s., nonsignificant.

Abbreviations: ADNI, Alzheimer's Disease Neuroimaging Initiative; NADS, Nanjing Aging and Dementia Study. P-MCI, progressive MCI, including MCI subjects who progressed to AD-type dementia at the three-year follow-up; N-MCI, non-progressive MCI, including MCI subjects who did not progress to dementia at the three-year follow-up; MCI, mild cognitive impairment; AD, Alzheimer's Disease; AVLT, Rey Auditory Verbal Learning Test; MMSE, Mini-Mental State Examination; HIP, hippocampus; PCC, posterior cingulate cortex; FUS, fusiform gyrus; CC, correlation coefficient.

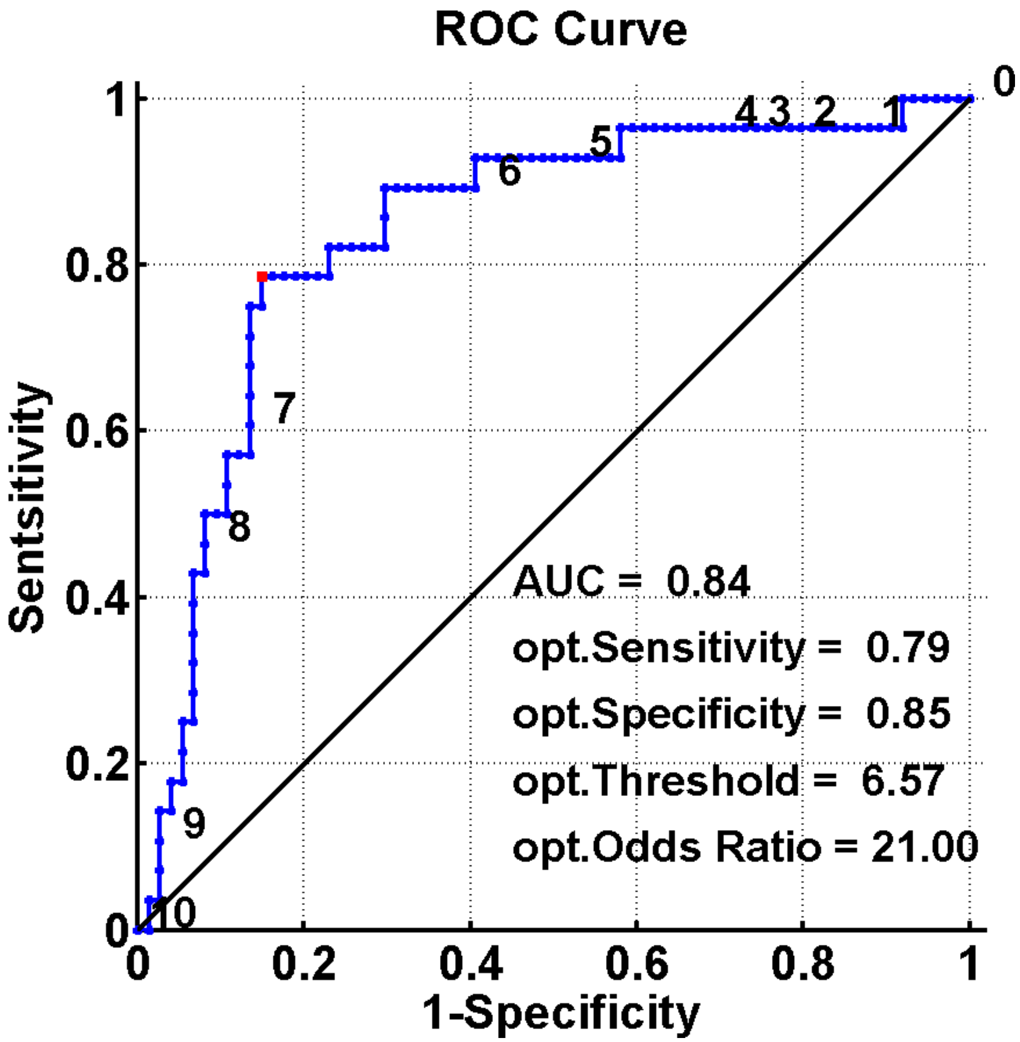

Figure S3, related to Figures 2 and 3. ROC curve of the CARE index stage in classifying the diagnosis of P-MCI versus N-MCI at baseline in combining ADNI and NADS datasets. Note: Numbers next to ROC curve indicate CARE index threshold. Abbreviations: ROC, receiver operating characteristic; AUC, area under curve; opt., optimum.

Supplementary References

1. Barros-Battesti DM, Ramirez DG, Sampaio Jdos S, Famadas KM, Faccini JL, Nunes PH et al. Description of larva of Amblyomma romitii (Acari: Ixodidae) by optical and scanning electron microscopy, including porotaxy and phylogenetic analysis. Experimental & applied acarology 2013; 60(2): 271-280.

2. McKhann G, Drachman D, Folstein M, Katzman R, Price D, Stadlan EM. Clinical diagnosis of Alzheimer's disease: report of the NINCDS-ADRDA Work Group under the auspices of Department of Health and Human Services Task Force on Alzheimer's Disease. Neurology 1984; 34(7): 939-944.

3. Petersen RC, Smith GE, Waring SC, Ivnik RJ, Tangalos EG, Kokmen E. Mild cognitive impairment: clinical characterization and outcome. Archives of neurology 1999; 56(3): 303-308.

4. Winblad B, Palmer K, Kivipelto M, Jelic V, Fratiglioni L, Wahlund LO et al. Mild cognitive impairment--beyond controversies, towards a consensus: report of the International Working Group on Mild Cognitive Impairment. Journal of internal medicine 2004; 256(3): 240-246.

5. Chen G, Chen G, Xie C, Ward BD, Li W, Antuono P et al. A method to determine the necessity for global signal regression in resting-state fMRI studies. Magnetic resonance in medicine : official journal of the Society of Magnetic Resonance in Medicine / Society of Magnetic Resonance in Medicine 2012; 68(6): 1828-1835.

6. Chen G, Shu H, Chen G, Ward BD, Antuono PG, Zhang Z et al. Staging Alzheimer's Disease Risk by Sequencing Brain Function and Structure, Cerebrospinal Fluid, and Cognition Biomarkers. Journal of Alzheimer's disease : JAD 2016; 54(3): 983-993.

7. Tzourio-Mazoyer N, Landeau B, Papathanassiou D, Crivello F, Etard O, Delcroix N et al. Automated anatomical labeling of activations in SPM using a macroscopic anatomical parcellation of the MNI MRI single-subject brain. NeuroImage 2002; 15(1): 273-289.

8. Xie W, Song C, Young NL, Sperling AS, Xu F, Sridharan R et al. Histone h3 lysine 56 acetylation is linked to the core transcriptional network in human embryonic stem cells. Mol Cell 2009; 33(4): 417-427.

9. Young AL, Oxtoby NP, Daga P, Cash DM, Fox NC, Ourselin S et al. A data-driven model of biomarker changes in sporadic Alzheimer's disease. Brain : a journal of neurology 2014; 137(Pt 9): 2564-2577.

10. Shu H, Shi Y, Chen G, Wang Z, Liu D, Yue C et al. Opposite Neural Trajectories of Apolipoprotein E 4 and 2 Alleles with Aging Associated with Different Risks of Alzheimer's Disease. Cerebral cortex 2016; 26(4): 1421-1429.

11. Chen J, Duan X, Shu H, Wang Z, Long Z, Liu D et al. Differential contributions of subregions of medial temporal lobe to memory system in amnestic mild cognitive impairment: insights from fMRI study. Scientific reports 2016; 6: 26148.

12. Chen J, Shu H, Wang Z, Liu D, Shi Y, Xu L et al. Protective effect of APOE epsilon 2 on intrinsic functional connectivity of the entorhinal cortex is associated with better episodic memory in elderly individuals with risk factors for Alzheimer's disease. Oncotarget 2016; 7(37): 58789-58801.
